# Lipid composition and abundance in the reproductive and alimentary tracts of female *Haemonchus contortus*

**DOI:** 10.1186/s13071-020-04208-w

**Published:** 2020-07-06

**Authors:** Tao Wang, Guangxu Ma, Shuai Nie, Nicholas A. Williamson, Gavin E. Reid, Robin B. Gasser

**Affiliations:** 1 Department of Veterinary Biosciences, Melbourne Veterinary School, Faculty of Veterinary and Agricultural Sciences, The University of Melbourne, Parkville, Victoria 3010 Australia; 2 Bio21 Mass Spectrometry and Proteomics Facility, The University of Melbourne, Parkville, Victoria 3010 Australia; 3 School of Chemistry, The University of Melbourne, Parkville, Victoria 3010 Australia; 4 Department of Biochemistry and Molecular Biology, The University of Melbourne, Parkville, Victoria 3010 Australia; 5 Bio21 Molecular Science and Biotechnology Institute, The University of Melbourne, Parkville, Victoria 3010 Australia

**Keywords:** *Haemonchus contortus*, Reproductive tract, Gut, Parasitic nematode, Lipidome, Lipids, Mass spectrometry, Adaptation

## Abstract

**Background:**

Lipids play essential structural and functional roles in the biology of animals. Studying the composition and abundance of lipids in parasites should assist in gaining a better understanding of their molecular biology, biochemistry and host-parasite interactions.

**Methods:**

Here, we used a combination of high-performance liquid chromatography and mass spectrometric analyses, combined with bioinformatics, to explore the lipid composition and abundance in the reproductive (Rt) and alimentary (At) tracts of *Haemonchus contortus*.

**Results:**

We identified and quantified 320 unique lipid species representing four categories: glycerolipids, glycerophospholipids, sphingolipids and steroid lipids. Glycerolipids (i.e. triradylglycerols) and glycerophospholipids (i.e. glycerophosphocholines) were the most commonly and abundant lipid classes identified and were significantly enriched in Rt and At, respectively. We propose that select parasite-derived lipids in Rt and At of adult female *H. contortus* are required as an energy source (i.e. triradylglycerol) or are involved in phospholipid biosynthesis (i.e. incorporated fatty acids) and host-parasite interactions (i.e. phospholipids and lysophospholipids).

**Conclusions:**

This work provides a first foundation to explore lipids at the organ-specific and tissue-specific levels in nematodes, and to start to unravel aspects of lipid transport, synthesis and metabolism, with a perspective on discovering new intervention targets.
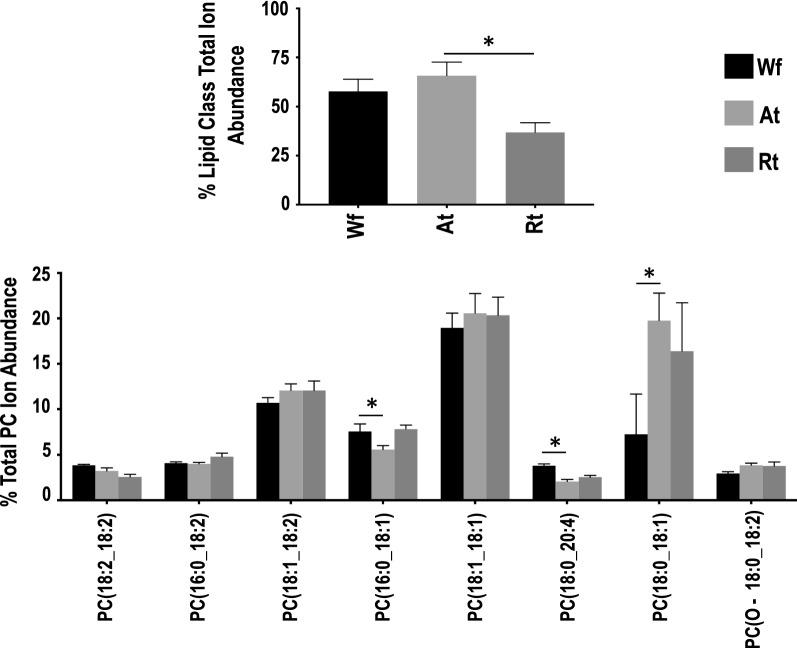

## Background

*Haemonchus contortus* (the barber’s pole worm) is one of the most pathogenic parasitic nematodes of ruminants, causing haemonchosis, which leads to major productivity and financial losses to agricultural and associated industries worldwide [1, 2]. Control relies heavily on chemotherapeutic treatment and/or vaccination [3]. However, due to the excessive and often uncontrolled usage of anthelmintics, *H. contortus* has developed resistance to most drug classes in current use [4], even to the recently-introduced amino-acetonitrile derivate, monepantel [5]. Despite the utility of vaccination in some age groups of animals (usually sheep) [1], there is a need to pursue the discovery of new interventions against haemonchosis. We believe that a profound understanding of the molecular biology of *H. contortus* could assist in the discovery of novel drug and vaccine targets in this worm.

In the past decade, progress has been made in the genomics, transcriptomics, proteomics, phosphoproteomics and lipidomics of this nematode via next generation nucleic acid sequencing or high throughput mass spectrometry approaches [6–13]. Studies are providing new and exciting insights into essential biological processes in key developmental stages of *H. contortus*, and are paving a way to identifying potential intervention targets, such as the bile acid-like hormone signalling receptors and cholesterol transporters (e.g. [14]). We believe that the study of lipids and their biology could also reveal intervention targets.

Recently, using a high throughput LC-MS/MS approach, we characterised the lipidome of different developmental stages and both sexes of *H. contortus*, and showed a substantial down-regulation of energy storage-related lipids, i.e. triradylglycerols (TG), during the transition from free-living to parasitic stages, suggesting critical and specific adaptations of the nematode (e.g. shifting or switching of nutrient acquisition) during its life-cycle [11]. Interestingly, TG abundance was relatively high in female adults of *H. contortus* compared with male adults. Considering the high abundance of total TG in eggs released by *H. contortus*, we proposed that immature eggs in the uterus represent a major proportion of TG in female worms. As nematodes, including *Ascaris*, convert enzymatically-released fatty acids into direct energy supply for egg development [15], it seems likely that *H. contortus* females actively accumulate energy as fat in embryos, to prepare them for life in the external environment. Logically extending previous work [15], we undertook the present study to gain a deeper insight into lipid composition and abundance in the reproductive and alimentary systems of the adult female of *H. contortus*.

## Methods

### *Haemonchus contortus* and its procurement

Adult worms of *H. contortus* were produced in sheep [16]. Worms were collected after 21 days of infection, washed five-times in large volumes (100 ml) of physiological saline (pH 7.0), and gravid female worms with a length of > 2 cm were selected. The reproductive (Rt) and alimentary (At) tracts were dissected from these female worms using a dissecting microscope (10× magnification), and snap-frozen at − 80 °C; whole gravid female worms (Wf; reference control) were frozen in the same manner. Four replicates were prepared for each Rt, At and Wf.

### Tissue homogenisation and lipid extraction

All four replicate samples of each Rt, At and Wf were individually homogenised and processed using an established method [11] and then lyophilised in a benchtop, manifold freeze-drier prior to extraction. Freeze-dried samples (2 mg each) were individually transferred to an Eppendorf tube (1.5 ml) containing 200 μl of ice-cold 40% (v/v) methanol. Samples were homogenised with 100 μl of 0.5 mm zirconium oxide beads (ZROB05, Next Advance, USA) in a blender (Bead Bullet, Next Advance, USA) twice for 3 min. Blank tubes with water (included as controls) were processed in the same manner. Lipids were extracted using an established method [17]. Briefly, 400 μl of chloroform/methanol (2/1, v/v) were added to each tube. The tubes were vortexed for 60 sec, and centrifuged at 3000×*g* and 22 °C for 10 min. The lower organic phase was then transferred to another tube, and 400 μl of 100% chloroform were added, vortexed (60 s) and centrifuged as before. The lower organic phase was collected and pooled with the organic phase from the first extraction. The pooled lipid samples were then dried in a SpeedyVac (4000× *g* for 45 min), and each lipid pellet was re-suspended in 10 μl isopropanol/methanol/chloroform (4/2/1, v/v/v) containing 0.01% butylated hydroxytoluene (BHT), and then diluted with 190 μl 100% methanol prior to analysis.

### High-performance liquid chromatography (HPLC) and mass spectrometric (MS) analyses

All three samples (four replicates for each) were analysed by ultrahigh performance liquid chromatography (UHPLC) coupled to tandem mass spectrometry (MS/MS) employing a Vanquish UHPLC linked to an Orbitrap Fusion Lumos mass spectrometer (Thermo Fisher Scientific, San Jose, CA, USA), with separate runs in positive and negative ion polarities. Solvent A was 6/4 (v/v) acetonitrile/water and solvent B was 9/1 (v/v) isopropanol/acetonitrile; both solvents A and B contained 10 mM ammonium acetate. Each sample (volume: 4 μl) was injected into an Accucore C30 column (2.1 × 250 mm, 2.6 µm; Thermo Fisher Scientific) at 40 °C at a flow rate of 350 μl/min for 1 min using 30% solvent B. During separation, the percentage of solvent B was increased from 30% to 70% in 5 min, from 70% to 93% in 9 min, from 93% to 99% in 7 min, and from 91% to 97% in 31 min. Subsequently, the percentage of solvent B was increased to 99.5% in 0.1 min and then maintained at 99.5% for 4.9 min. Finally, the percentage of solvent B was decreased to 30% in 0.1 min and maintained for 3.9 min.

All MS experiments were performed using a Heated Electrospray Ionization (HESI) source. The spray voltages were 3.5 kV in positive ionisation-mode and 3.0 kV in negative ionisation-mode. In both polarities, the flow rates of sheath, auxiliary and sweep gases were 20 and 6 and 1 ‘arbitrary’ units, respectively. The ion transfer tube and vaporizer temperatures were maintained at 350 °C and 400 °C, respectively, and the S-Lens RF level was set at 50%. In the positive ionisation-mode from 1 to 28 min, top speed data-dependent scan with a cycle time of 1 s was used. Within a cycle, a full-scan MS spectra were acquired firstly in the Orbitrap at a mass resolving power of 120,000 (at m/z 200) across an m/z range of 300–2000 using quadrupole isolation, an automatic gain control (AGC) target of 4e5 and a maximum injection time of 50 msec. Then, every higher-energy collisional dissociation (HCD)-MS/MS was performed in the cycle at a mass resolving power of 15,000 (at m/z 200), a normalised collision energy (NCE) of 27%, an m/z isolation window of 1, a maximum injection time of 35 milliseconds and an AGC target of 5e4. For the improved structural characterisation of glycerophosphocholine (PC) lipid ions, a data-dependent product ion (m/z 184.0733)-triggered collision-induced dissociation (CID)-MS/MS scan was performed in the cycle using a q-value of 0.25 and a NCE of 30%, with other settings being the same as that for HCD MS/MS. For the improved structural characterisation of TG lipids, the fatty acid + NH3 neutral loss product ions observed by HCD-MS/MS were used to trigger the acquisition of the top-3 data-dependent CID-MS3 scans in the cycle using a q-value of 0.25 and a NCE of 30%, with other settings being the same as that for HCD MS/MS.

### Identification and quantification of lipids and statistical analysis

MS data were processed using LipidSearch software v.4.2.23 (Thermo Fisher Scientific, San Jose, CA, USA) [18]. Key processing parameters were: target database, general; precursor tolerance, 5 ppm; product tolerance, 5 ppm; product ion threshold, 1%; m-score threshold, 2; quantification m/z tolerance, ± 5 ppm; quantification retention time range, ± 1 min; use of main isomer filter and ID quality filters A, B and C; adduct ions, +H and +NH4 for positive ionisation mode, and −H and +CH3COO for negative ionisation mode. All lipid classes available were selected for the search. The same lipid annotations (within ± 0.1 min) were merged into the aligned results. Unassigned peak areas were calculated for relative quantification and alignment. The shorthand notation used for lipid classification and structural representation follows the nomenclature proposed previously [19, 20]. To filter false identifications, aligned lipids were examined manually. For TG lipids, a grade A-identification required at least one replicate of each sample, whereas a grade A-, B- or C-identification required only monoacylglycerol (MG) lipids. For all other lipid classes, a grade A- or B-identification required at least one replicate of each sample. For all lipids, a signal-to-noise ratio (S/N) > 10 and a peak area > 1e7 were required in at least one sample group. All lipid LC-MS features were manually inspected and re-integrated when needed. To compare the relative abundance of lipids between tracts (tissues) and the whole worm of *H. contortus*, the ion abundances of corresponding lipid categories and classes were expressed as a percentage of the total ion abundance for the total lipidome, whereas the ion abundances of corresponding lipid species were expressed as a percentage of the total ion abundance for a particular lipid class. Venn diagrams of individual lipidomes were produced using the R package v.3.3.1. One-way ANOVA with *post-hoc* multiple comparison tests was performed using GraphPad Prism 8.2.1 software (GraphPad, La Jolla, USA). Error bars represent the relative standard deviation of the mean (RSD). Statistical significance was set at *P* < 0.05.

## Results and discussion

### Lipid identification

**Fig. 1:**
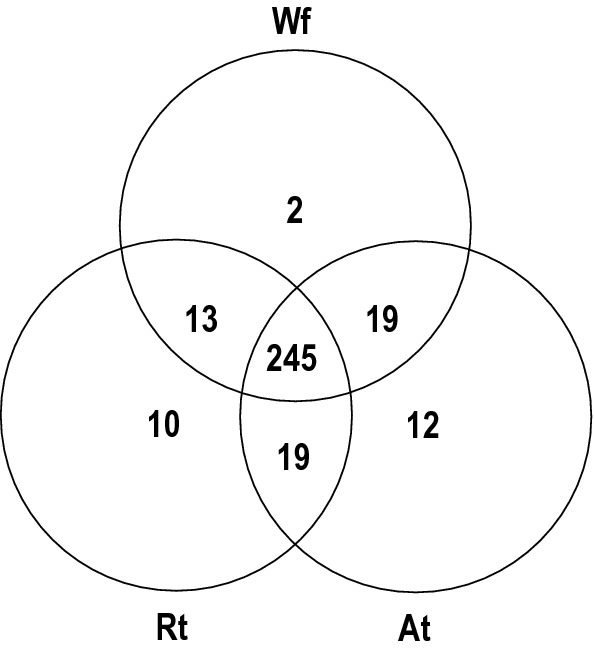
Venn diagram showing the similarity of lipid species identified in extracts derived from whole adult female (Wf) worms of *Haemonchus contortus* and their reproductive (Rt) and alimentary (At) tracts

The LC-MS/MS-based lipidomic analysis of lipid extracts (four replicates each) from whole adult female (Wf) worms of *H. contortus* and their reproductive (Rt) and alimentary (At) tracts allowed the identification and quantification of a total of 320 unique lipid species, belonging to four lipid categories (i.e. glycerolipids (GL), glycerophospholipids (GP), sphingolipids (SP) and sterol lipids (ST)) and 18 lipid classes (Table 1 and Additional file 1: Table S1). Consistent with the available global lipidome data set [11], lipid species from GL and GP categories were most commonly identified in all of the samples. Lipids from these two classes contributed to ~ 90% of the lipid species identified, and TG was the most-commonly identified lipid class (*n* = 236), followed by glycerophosphoethanolamine (PE; *n* = 50) and glycerophosphocholine (PC; *n* = 31). By contrast, only small numbers of lipid species were found to represent the glycerophosphoglycerols (PG; *n* = 2) and lyso-glycerophosphoglycerols (LPG; *n* = 3) (Table 1). The number of lipid species shared among Rt, At and Wf samples are displayed in a Venn diagram (Fig. 1). Most of the lipid species (*n* = 296, 92.5%) were commonly detected in at least two of the samples analysed. Most lipid species (*n* = 245, 76.6%) were detected in Wf, Rt and At, whereas 2, 10 and 12 lipid species were unique to each of these samples, respectively. The list of lipid species identified in individual samples is given in Additional file 1: Table S1.

**Table 1:** Summary of the numbers of identified lipid species in extracts derived from whole adult female (Wf) worms of *Haemonchus contortus* and their reproductive (Rt) and alimentary (At) tracts

Lipid category/class	Wf	Rt	At	Total no. of lipids identified
Glycerolipids				
MG	3	2	0	4
DG	17	11	15	19
TG	88	94	94	107
Glycerophospholipids				
PC	50	49	50	50
PE	30	31	30	31
PG	2	2	2	2
PI	17	19	18	19
PS	4	5	4	5
CL	8	8	8	8
LPC	15	16	16	16
LPE	11	11	11	11
LPG	3	2	2	3
LPI	5	5	5	5
LPS	7	7	7	7
Sphingolipids				
SM	4	4	3	4
Cer	19	19	12	19
HexCer	4	7	2	7
Sterol lipids				
CE	0	3	0	3
In total	287	295	279	320

*Abbreviations*: MG, monoradylglycerols; DG, diradylglycerols; TG, triradylglycerols; PC, glycerophosphocholines; PE, glycerophosphoethanolamines; PG, glycerophosphoglycerols; PI, glycerophosphoinositols; PS, glycerophosphoserines; CL, cardiolipins; SM, sphingomyelins; Cer, ceramide; HexCer, hexosylceramide; CE, cholesteryl ester

*Note*: For LPC, LPE, LPG, LPI and LPS, prefix “L” was added for each lysoglycerophospholipid class

### Lipid quantification

**Fig. 2:**
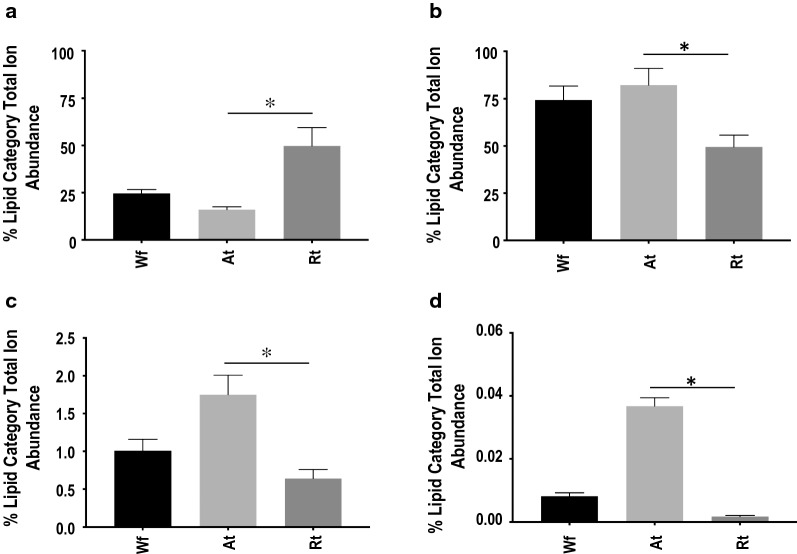
Relative quantification changes of lipid categories from whole adult female (Wf) worms of *Haemonchus contortus* and their reproductive (Rt) and alimentary (At) tracts. **a** Glycerolipids. **b** Glycerophospholipids. **c** Sphingolipids. **d** Sterol lipids. Statistical analysis was performed by ANOVA (*P < 0.05; see Additional file 2: Table S2 for details). Error bars indicate ± RSD (four replicates)

**Fig. 3:**
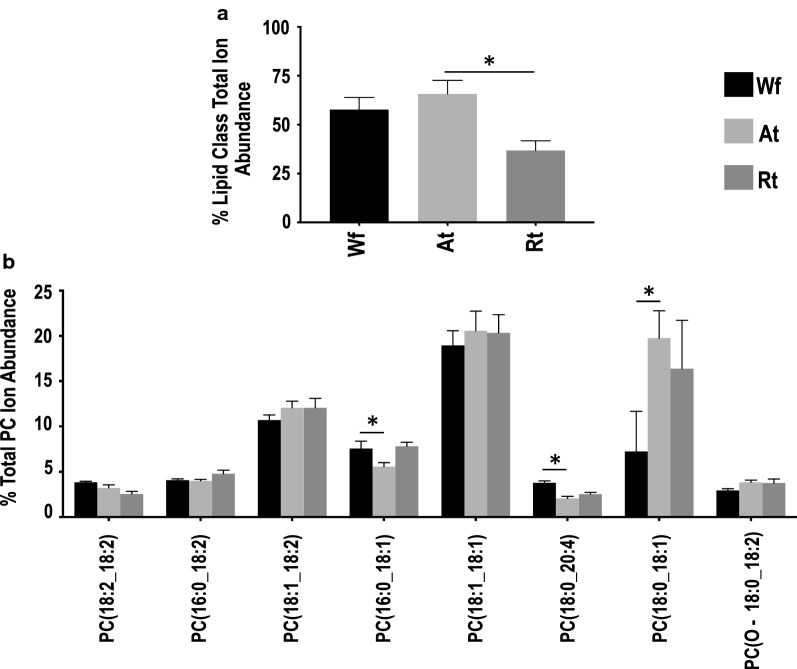
Relative quantification changes of total (**a**) and individual (**b**) PC lipids from whole adult female (Wf) worms of *Haemonchus contortus* and their reproductive (Rt) and alimentary (At) tracts. Only individual lipid species with > 4% ion abundance (at any life stage) are shown. Statistical analysis was performed by ANOVA (*P < 0.05; see Additional file 2: Table S2 for details). Error bars indicate ± RSD (four replicates)

**Fig. 4:**
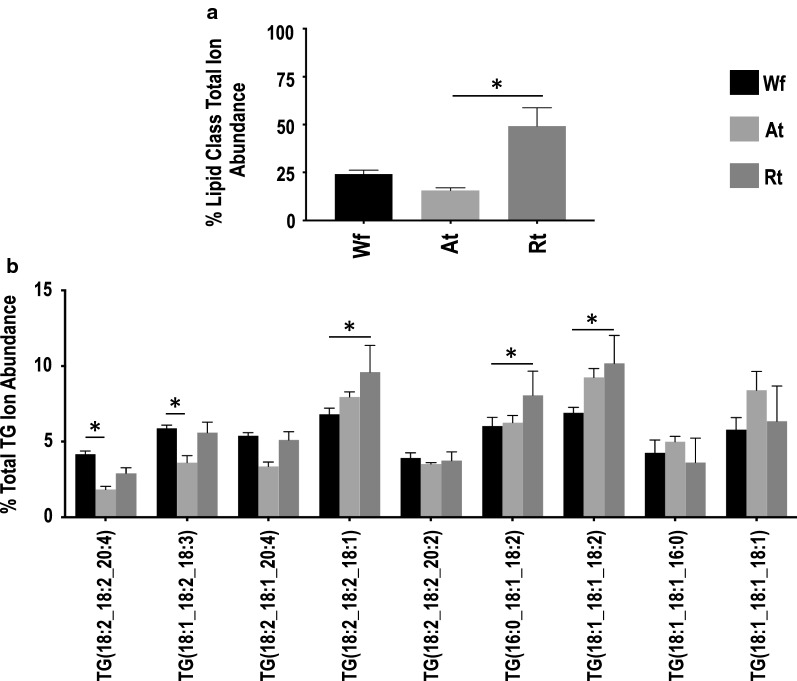
Relative quantification changes of total (**a**) and individual (**b**) TG lipids from whole adult female (Wf) worms of *Haemonchus contortus* and their reproductive (Rt) and alimentary (At) tracts. Only individual lipid species with > 4% ion abundance are shown. Statistical analysis was performed by ANOVA (*P < 0.05; see Additional file 2: Table S2 for details). Error bars indicate ± RSD (four replicates)

Similar to the Wf lipidome [11], relative quantification analysis revealed that GP (49.6–82.1%) and GL (16.1–49.8%) were the two most abundant lipid categories in *H. contortus* at the organ system-level (i.e. in both Rt and At), whereas lipids from SP and ST categories contributed < 2% to the total ion abundance in the lipidome of *H. contortus* (Fig. 2). Within the GP category, membrane structure-related PC (37–66%) and PE (10.5–11.3%) were the major classes. Notably, a significantly lower level of PC was measured in Rt compared with At (Fig. 2; Additional file 2: Table S2), although there was no significant difference for PE. Further analysis of individual lipid species showed that PC lipids with even-numbered fatty acyl chains (e.g. 18:0, 18:1 and 18:2) and long fatty acyl chains (> 34 total carbons) predominated (Fig. 3). In contrast, the energy storage-related TG (15.6–49.2%) was the most abundant class in the GL category and had a significantly higher abundance in Rt than At (Fig. 4; Additional file 2: Table S2). Interestingly, TG species, such as TG (18:2_18:2_18:1) and TG (16:0_18:1_18:2), contributed predominantly to this abundance difference between the two organ systems (tracts), whereas TG (18:1_18:1_18:1) did not. Similar to PC, individual lipid species with even-numbered fatty acyl chains (e.g. 18:1, 18:2 and 20:4) and long fatty acyl chain (> 50 total carbons)-fatty acids were the main representatives of the TG class. No significant difference in abundance was detected for lipid classes PG and LPG, both of which had a relatively low quantification level.

### Lipid composition

**Fig. 5:**
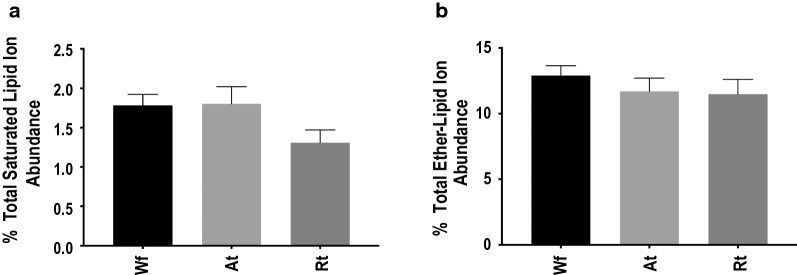
Relative quantification changes of (**a**) total saturated fatty acyl- and (**b**) ether-linked lipid ion abundance in the whole adult female (Wf) worms of *Haemonchus contortus* and their reproductive (Rt) and alimentary (At) tracts. Error bars indicate ± RSD (four replicates)

The analysis of fatty acyl compositions showed that lipids of *H. contortus* contain a high level of unsaturated, even-numbered and long-chain fatty acyls (> 12 carbons), which represented 70.0%, 87.9% and 97.5% of the total fatty acyl composition, respectively (Table 2). The identified saturated lipid species were found mainly in ceramide (Cer, *n* = 8), hexosylceramide (HexCer, *n* = 4) and LPG (*n* = 4). In addition, 47 ether-linked lipid species were identified; they were mainly in the GP category (*n* = 42), representing particularly the classes PE (*n* = 18) and PC (*n* = 9), whereas the other 5 ether-linked lipids represented the TG class. Nonetheless, no significant difference in the total ion abundance was detected for saturated or ether-linked lipids among three samples for each Wf, Rt and At (Fig. 5).

**Table 2:** Fatty acyl (FA) composition of identified lipid species in the lipidome of the adult female of *Haemonchus contortus*

Lipid category	Saturated (%)		Unsaturated (%)		Odd-numbered chain FA (%)	Even-numbered chain FA (%)	Total no. of FAs
	Medium-chain FA^a^	Long-chain FA^b^	Medium-chain FA	Long-chain FA			
Glycerolipids	2.5	11.4	nd	37.0	5.2	45.7	796
Glycerophospholipids	nd	10.9	nd	29.3	2.2	38.0	413
Sphingolipids	nd	5.2	nd	3.2	4.6	3.8	78
Steroid lipids	nd	0	nd	0.4	nd	0.4	11
In total	2.5	27.5	0	70.0	12.1	87.9	1298

^a^Medium-chain FA contains 6–12 carbons

^b^Long-chain FA contains > 12 carbons

*Abbreviation*: nd, not detected

### Proposal that particular lipids are required for parasite adaptation, energy storage and development

The constantly changing external environments force parasitic worms to adapt their nutrient acquisition and metabolism [21–23]. Previously, we observed substantial alterations in the lipidome of *H. contortus*, in terms of lipid composition and abundance, during the transition from free-living to parasitic stages, indicating that energy metabolism was linked to adaptation in this nematode [11]. Conspicuously, the energy storage-related triradylglycerols (TG) abundance was relatively high in adult female worms of *H. contortus*.

The significantly higher abundance level of TG lipids in the reproductive system of the female worm compared with the gut indicated that the eggs in the uterus likely contribute most to the TG proportion in gravid female worms. Given that fatty acid beta-oxidation does not occur in the adult stage of parasitic worms of animals [21, 24], they are not able to consume exogenous lipids as a direct energy source. Evidence indicates that adult worms tend to modify (e.g. elongate and desaturate) as well as accumulate these incorporated fatty acids, mainly in the format of TG, into embryos for further development [21, 24]. On the other hand, it has been shown that early egg and larval developmental stages of parasitic worms (e.g. *H. contortus* and *Ascaris lumbricoides*) can convert endogenous lipids acquired maternally from a previous host into carbohydrates (i.e. two acetyl-CoA units into succinate and malate) *via* beta-oxidation and glyoxylate cycle [25, 26]. These endogenous lipids are believed to be an energy source (of predominantly TG) to prevent worm starvation and are building blocks (e.g. phospholipids) for the biosynthesis of membranes [24, 27–29]. Intriguingly, Bexkens et al. [24] observed a decrease in the number and size of lipid droplets (i.e. containing mainly TG) and an increase in cellular membrane lipids during egg development in the parasitic blood-fluke *Schistosoma mansoni*, suggesting that endogenous lipid stores are used for phospholipid biosynthesis. Based on a previous observation of lipid composition and abundance in eggs and third-stage larvae (L3s) of *H. contortus* [11], it is reasonable to propose that similar biosynthesis occurs during egg development in *Haemonchus*. It would be interesting to undertake a detailed ‘time-course’ lipidomic study of synchronised *Haemonchus* eggs during their development to larvae, in order to test this hypothesis.

### Parasite-derived lipids could play important roles in host-parasite interactions

**Fig. 6:**
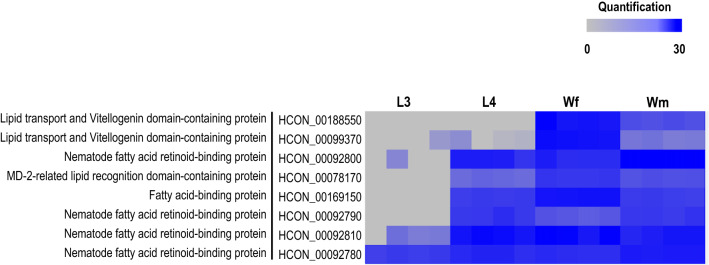
The display of the quantification changes of lipid binding associated proteins in the secretome of different developmental stages/sexes (i.e. third-stage (L3), fourth-stage (L4) larvae and whole adult female (Wf) and male (Wm) worms of *H. contortus* (see [12]). Normalised protein abundance (columns) is shown in a grey-to-blue scale, depicting a low to high protein abundance

In addition to the biochemical roles of lipids in energy storage and membrane synthesis, it is becoming increasingly evident that parasite-derived lipids play important roles in host-parasite interactions, and that phospholipids (e.g. PC and PE) and lysophospholipids (e.g. LPC and LPS) are precursors for the regulation of various essential signalling pathways in animals [30]. Previous biochemical studies of tegumental lipids of *S. mansoni* [27] have provided initial insight into the central roles that parasite-derived phospholipids and lysophospholipids play in lipid-associated host-parasite interactions. For instance, it has been shown that schistosome tegument-specific LPS and LPC species are able to downregulate host immune-modulation, leading to a decrease in eosinophil activation and cytokine production, *via* the stimulation of Toll-like receptor-2-dependent mechanisms in the immune system of the host animal [28, 31, 32]. Moreover, using direct infusion quadrupole time-of-flight (Q-TOF) mass spectrometry, some parasite-derived ether-linked phospholipids (i.e. PC and PE) were discovered in the fluid from *Onchocerca ochengi*-induced nodules in cattle [33], indicating a release of these molecules into host tissues. In the present study, substantial phospholipids (e.g. PC) were enriched in the gut of *H. contortus*, including ether-linked phospholipids (e.g. PC E-34:2, PC E-36:2 and PC E-38:2) (Fig. 3 and Additional file: Table S1). Unlike the lipid-enriched tegument of flatworms, which transports nutrients into the worm and by-products out of the worm [27], the surface of parasitic nematodes is covered by a tough and almost impermeable cuticle, composed primarily of proteins and a limited amount of lipids [34]. This means that the nematode intestine is likely the primary source of worm-derived lipids, released for cross-talk between worm and host. Although there is presently no direct evidence, it seems reasonable to propose that nematode-derived glycerophospholipids are released directly from nematode intestine, possibly requiring lipid transfer or transport proteins. Interestingly, in a previous study of the developmental secretome of *H. contortus*, we identified a panel of lipid binding-associated proteins (*n* = 8), such as fatty acid and retinoid-binding proteins and vitellogenin (egg-yolk protein) [10]. These proteins were believed to be involved in lipid transportation [34]. Evidence that such proteins are excreted/secreted mainly by parasitic stages (i.e. L4, adult female and adult male), rather than free-living L3 stage (Fig. 6), suggests heavy demands on the use of these proteins as vehicles for worm-derived lipid delivery to the host as *Haemonchus* establishes in the stomach (abomasum) of its host animal.

## Conclusions

This high-throughput LC-MS/MS-based lipidomic study has inferred that a spectrum of *H. contortus* lipids is likely required as an energy source, for development and adaptation and/or for the maintenance of an intricate host-parasite cross-talk. Finding ways of disrupting or interrupting this interplay has the potential to lead to new intervention strategies.

## Supplementary information

**Additional file 1: Table S1.** Relative quantification changes of total saturated fatty acids and ether-linked lipid ion abundance from whole adult female (Wf) worms of *Haemonchus contortus* and their reproductive (Rt) and alimentary (At) tracts.

**Additional file 2: Table S2.** Results of statistical analyses (one-way ANOVAs).

## Data Availability

Data supporting the conclusions of this article are included within the article.

## Abbreviations

Wf: whole adult female
Rt: reproductive tract
At: alimentary track
MG: monoradylglycerols
DG: diradylglycerols
TG: triradylglycerols
PC: glycerophosphocholines
PE: glycerophosphoethanolamines
PG: glycerophosphoglycerols
PI: glycerophosphoinositols
PS: glycerophosphoserines
CL: cardiolipins; For LPC, LPE, LPG, LPI and LPS, prefix “L” was added for each lysoglycerophospholipid class
SM: sphingomyelins
Cer: ceramide
HexCer: hexosylceramide
CE: cholesteryl ester
FA: fatty acyl
RSD: relative standard deviation
